# Visible-light-driven NHC and organophotoredox dual catalysis for the synthesis of carbonyl compounds

**DOI:** 10.3762/bjoc.21.200

**Published:** 2025-11-21

**Authors:** Vasudevan Dhayalan

**Affiliations:** 1 Department of Chemistry, National Institute of Technology Puducherry, Karaikal-609609, Puducherry, India

**Keywords:** dual catalysis, NHC, organic photocatalyst, radicals, visible-light

## Abstract

Over the past two decades, organocatalyzed visible-light-mediated radical chemistry has significantly influenced modern synthetic organic chemistry. In particular, dual catalysis combining *N*-heterocyclic carbenes (NHCs) with organophotocatalysts (e.g., 4CzIPN, eosin Y, rhodamine, 3DPAFIPN, Mes-Acr-Me^+^ClO_4_^−^) has emerged as a powerful photocatalytic strategy for efficiently constructing a wide variety of carbonyl compounds via radical cross-coupling processes. This cooperative organic dual catalysis has great potential in medicinal, pharmaceutical, and materials science applications, including the development of organic semiconductors and polymers. In recent years, NHC-involved photocatalysis has attracted considerable attention in synthetic organic chemistry, and particularly in the late-stage functionalization of bioactive compounds, drugs, and natural products. This review highlights recent advances in NHC–organophotoredox dual catalysis, focusing on methodology development, mechanistic insights, and reaction scope for synthesizing carbonyl compounds and pharmaceutically relevant intermediates. Moreover, this catalytic system operates under green and sustainable conditions, tolerating a broad range of functional groups and substrate scope, and utilizes low-cost, atom-economical, non-toxic starting materials.

## Introduction

Over the last ten years, NHC-catalyzed visible-light-promoted radical chemistry has been extensively developed for the cost-effective and practical synthesis of bioactive intermediates, pharmaceuticals, drugs, and natural products [1–6]. Recently developed photocatalysis affords sustainable, regioselective green methods for producing a wide range of functionalized carbonyl compounds and their related bioactive chiral intermediates under mild conditions, employing dual organic photoredox catalysis [7–11]. The use of visible light as an energy source has significantly expanded the scope of organic molecule activation and its application in organic synthesis or medicinal chemistry [12], thereby driving the fast development of various photocatalytic transformations. Among these advances, dual catalysis, particularly the synergistic combination of *N*-heterocyclic carbenes (NHCs) with organic photocatalysts, has opened new avenues in molecular construction, particularly for the novel, practical preparation of carbonyl group-containing compounds [13–16]. These ubiquitous ketone, ester, and amide functional groups are found in various drugs, natural products, and optoelectronic materials.

In the last three decades, *N*-heterocyclic carbenes (NHCs) have been renowned as versatile organocatalysts, including thiazolium, imidazolium, and triazolium moieties. NHCs are extensively used in many catalytic asymmetric reactions and are functional transformations in synthetic organic chemistry. Especially, 1,2,3-triazole-based NHCs are generally more reactive and stronger σ-donors than imidazole or thiazole analogues. Triazolium NHC enhances their ability to stabilize reactive radical intermediates or acyl anion equivalents, which is crucial in dual or triple catalytic cycles. The electron-rich carbene center facilitates faster SET processes in photocatalytic reactions. Triazolium-based NHCs exhibit higher oxidative and photochemical stability, making them well-suited for visible-light-driven catalytic transformations. Previous reports displayed various research groups successfully designed and prepared a variety of triazolium-based NHCs, eventually leading to the development of chiral NHC scaffolds by Knight and Leeper [17], followed by the remarkable contributions of Bode [18], Rovis [19], Glorius [20], Enders [21], Rafinski [22], Scheidt [23], Connon [24], Chi [25], Milo [26–27], Gravel [28–29] and others [30]. These NHC-catalyzed developments have drastically improved the achievable diastereo- and enantioselectivity, including common named reactions such as benzoin reactions, Stetter reactions, Michael additions, cycloadditions, domino reactions, cascade annulations, Diels–Alder reactions, and Michael–Stetter reactions, to name a few [31–35].

Notably, previous reports have demonstrated that the utility of chiral *N*-heterocyclic carbene (NHC) catalysts permits contracting asymmetric C–C, C–HA bond-forming reactions [36–37]. These chiral NHC catalysts, used to access enantiopure alcohol/amine derivatives, particularly 2° and 3° alcohols/amines, are significant structural motifs in numerous drugs and natural products and have found widespread synthetic applications in medicinal chemistry and active pharmaceutical ingredients [38–40].

Over the years, the groups of Hopkinson, Wang, Dong, Marzo, and co-workers published a comprehensive review detailing the synthetic approaches and mechanistic insights of visible light-promoted dual photocatalysis that combined *N*-heterocyclic carbenes (NHCs) with photocatalysts. The review encompasses transition-metal-based photocatalytic reactions for C–C and C–HA cross-coupling reactions involving various acyl fluorides, amides, aldehydes, carboxylic acids, and esters, highlighting their broad applications in organic synthesis and medicinal chemistry. However, to my knowledge, a review paper on visible-light-promoted dual organocatalyzed photochemical reactions has not been reported. Therefore, this work is helpful for readers to gain a comprehensive understanding of NHC-based dual organocatalyzed photochemical reactions [13–16]. The groups of MacMillan, Studer, Chi, Plunkett, Rovis, Hopkinson, and others developed NHC-involved radical reactions via visible light-promoted dual or triple photocatalysis, including NHC and transition metal-based photocatalytic reactions and their application in organic synthesis [41–50].

This review focuses on recent synthetic developments of NHC-catalyzed, visible-light-promoted dual organophotocatalysis for preparing carbonyl compounds and related organic intermediates by combining NHC and organic photocatalysts. Organocatalysts have a significant impact on the process, such as being environmentally friendly, offering operational simplicity, lower cost, and easy availability, reducing waste and toxicity, as well as providing excellent chemo-, regio-, and enantioselectivity through fine-tuned hydrogen bonding or covalent activation modes. Furthermore, the continued integration of this versatile photocatalytic platform with sustainable methods such as visible-light harvesting and the use of earth-abundant catalysts is expected to further enhance its impact across diverse research fields, including chemical biology, pharmaceuticals, agrochemicals, polymers, and advanced optical and energy materials.

## Review

### Visible-light-driven NHC/4CzIPN-catalyzed reactions

**Scheme 1:**
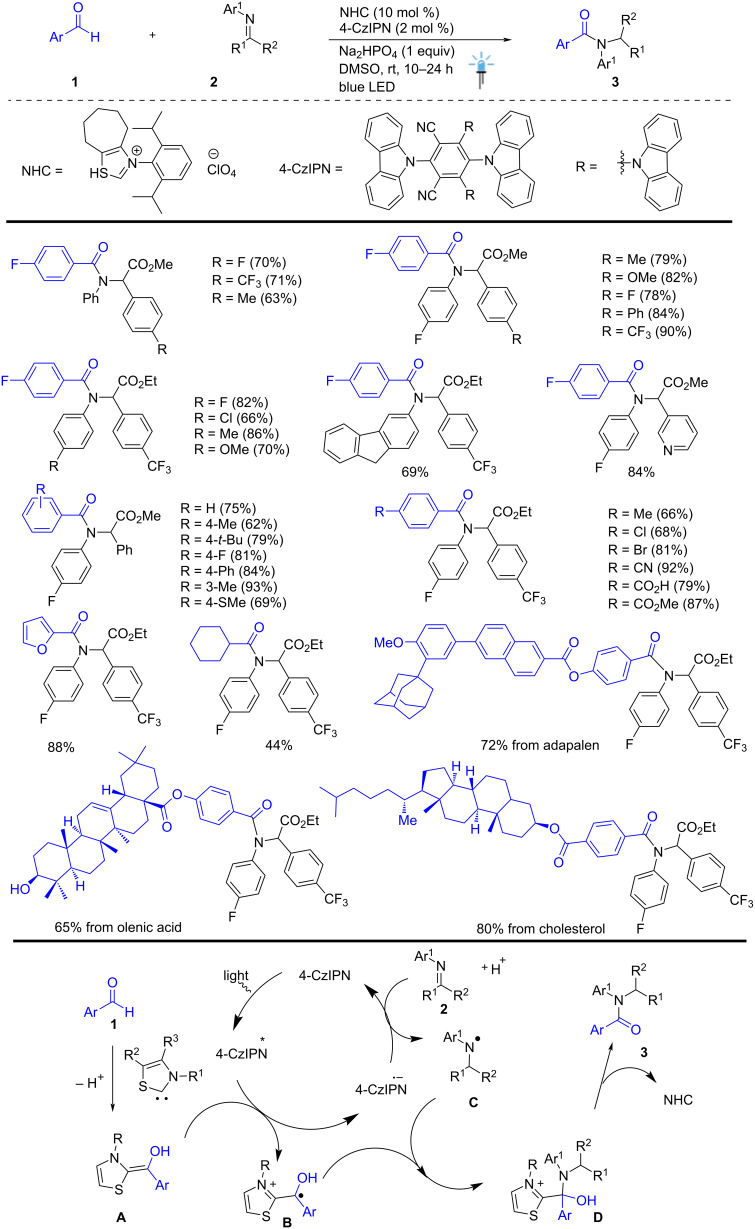
NHC-catalyzed umpolung strategy for the metal-free synthesis of amide via dual catalysis.

Recently, Shu and co-workers developed a direct and innovative preparation of highly functionalized aryl amide derivatives **3** from aryl aldehydes **1** and substituted imines **2** under mild conditions in the presence of NHC (10 mol %) and 4CzIPN (2 mol %) and Na_2_HPO_4_ in DMSO at rt for 10–24 h. The key to success lies in the photocatalytic dual system, which combines two organocatalysts (NHC/4CzIPN) and visible light irradiation to permit a novel umpolung single-electron reduction of respective imino ester **2**, generating an N-centered radical species **C**. This species subsequently undergoes a rapid C–N cross-coupling with ketyl radical **B**. This cross-coupling method offers a transition-metal free route to highly substituted amides **3** from aldehydes **1** and imines **2**, without the need for any external reductants or oxidants, and proceeds in an atom-economical manner. This photochemical reaction displays a wide substrate scope, tolerating a broad range of aryl and aliphatic aldehydes with good functional group compatibility. Mechanistic studies revealed that the photochemical reaction proceeds via radical–radical cross-coupling between the C-centered and N-centered radicals. These methods are expected to open new avenues for visible-light- and NHC/4CzIPN-catalyzed carbon–heteroatom bond-forming processes (Scheme 1) [51].

**Scheme 2:**
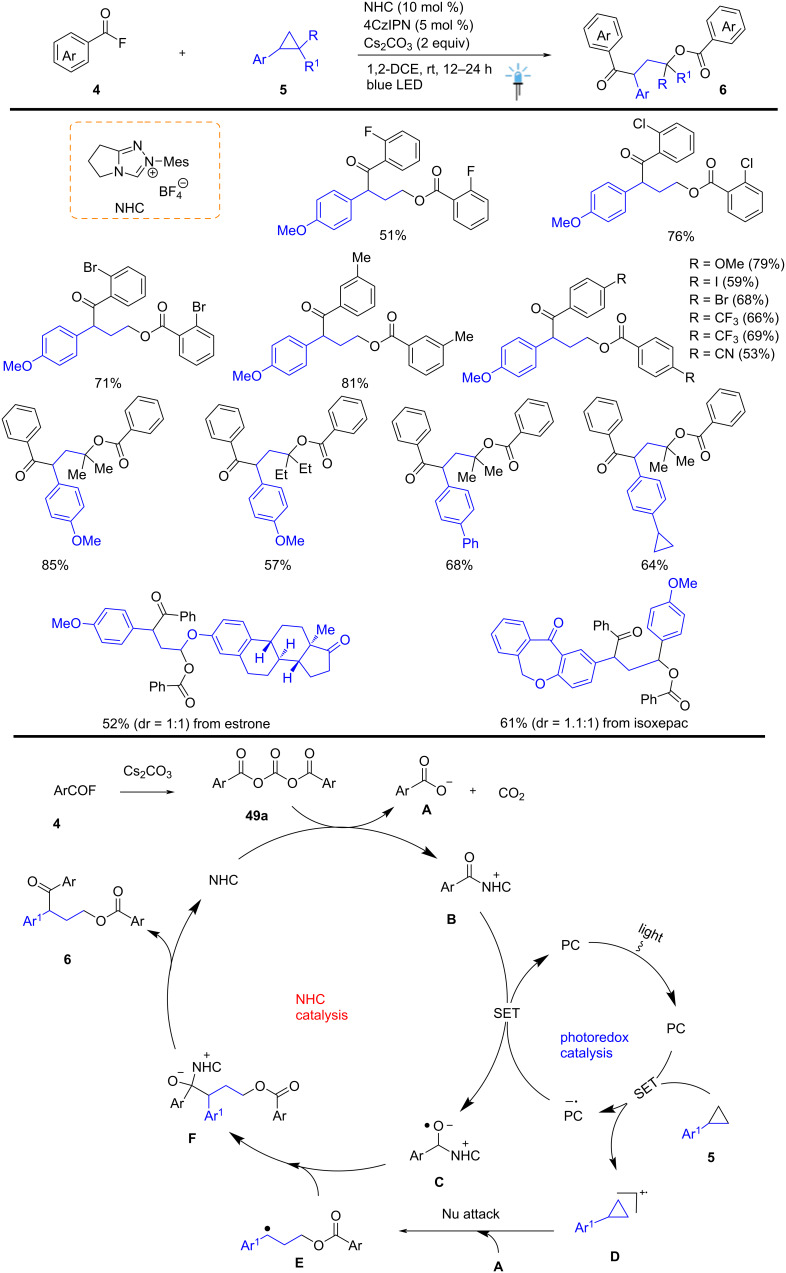
Visible-light promoted cooperative NHC/photoredox catalyzed ring-opening of aryl cyclopropanes.

In 2021, Studer et al. developed a novel method for the 1,3-difunctionalization of arylcyclopropanes **5** by applying NHC/ photoredox cooperative organocatalysis under visible-light irradiation. This method allows sequential C–O and C–C bond formation, leading to access to various γ-aroyloxy keto-ester derivatives **6** in good yield up to 81% and excellent functional group tolerance, including electron-rich and electron-poor substituents. This reaction was carried out between arylcyclopropanes **5** and acyl fluoride **4** in the presence of NHC (10 mol %) and 4CzIPN (5 mol %). Mechanistic studies showed that the cascade proceeds via nucleophilic ring-opening of a cyclopropyl radical cation **D** with subsequent radical-/radical cross-coupling performed between intermediates **C** and **E**. The reaction of acyl fluoride **4** in the presence of Cs_2_CO_3_ and NHC to produce benzoate **A** and the formation of some reactive intermediates are shown in Scheme 2. These methods will find suitable application in medicinal chemistry and related biological branches (Scheme 2) [52].

**Scheme 3:**
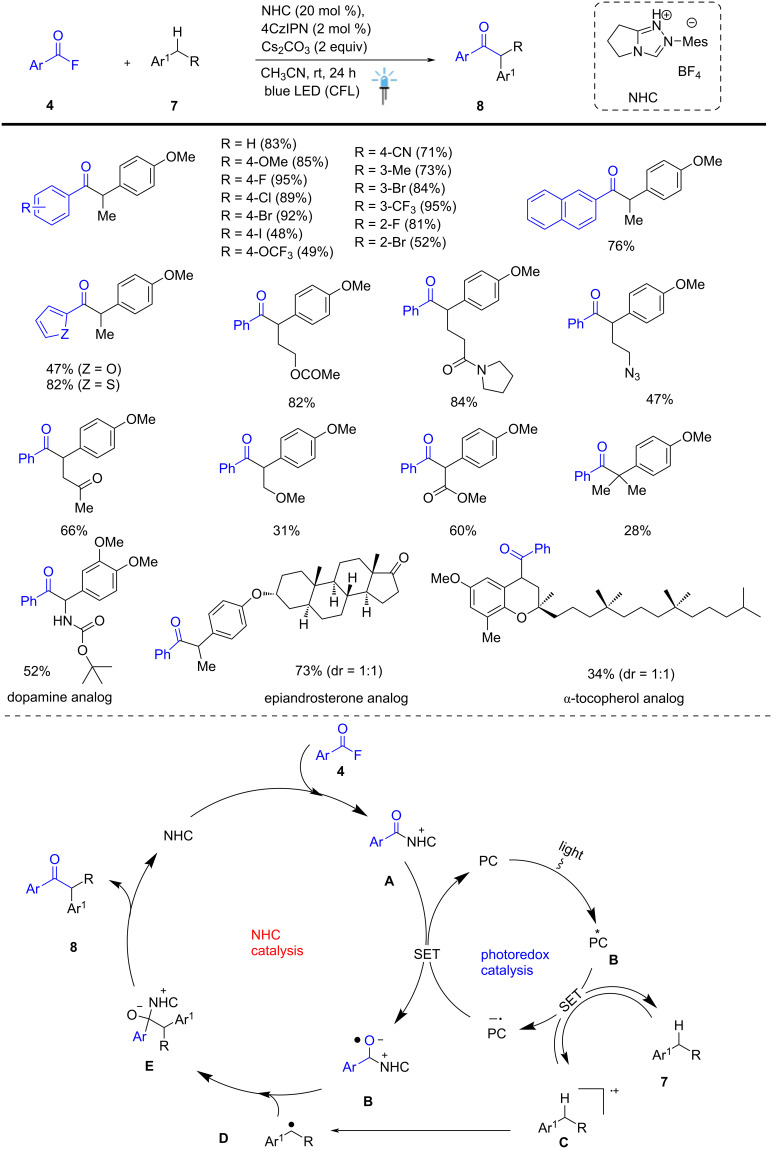
NHC-catalyzed benzylic C–H acylation by dual catalysis.

Studer and his group efficiently developed a method to achieve the acylation of benzylic C(sp³)–H bonds via cooperative NHC and organophotocatalyzed dual catalysis. The key step in the reaction is a radical–radical cross-coupling process leading to the production of ketone scaffolds, including drug moieties. The organocatalyzed acylation proceeds with excellent site selectivity and broad functional group tolerance of alkanes such as F, Cl, Br, I, CN, CF_3_, Me, and OMe. The reaction was achieved between acyl fluoride **4** and highly substituted alkane **7** in the presence of NHC (20 mol %), 4CzIPN (2 mol %) under mild conditions, producing corresponding unsymmetrical ketone derivatives **8** in up to 95% yield**.** An Ir-based photocatalyst was initially selected because its excited state is a strong oxidant (*E*_1/2_[Ir*^III/II^] = +1.21 V). Although 4-ethylanisole exhibits a higher oxidation potential (*E*_1/2_ = +1.52 V). This reaction was examined using different solvents, and it was found that toluene and 1,4-dioxane gave low yields (4–5% yield). Under blue LED irradiation, Ir-based PC is photoexcited, and its excited state is reductively quenched by the electron-rich arene substrate **7**, generating the aryl radical cation **C** along with the formation of corresponding radical anion of the photocatalyst (PC^•–^). The reduction potentials are (*E*_1/2_(P/P^•–^) = –1.37V vs SCE for [Ir(dF(CF₃)ppy)₂(dtbbpy)]PF₆ and –1.21V vs SCE for 4CzIPN as an organophotocatalyst. This method permits the C–H bond functionalization of important structural motifs with good to excellent diastereoselectivities, high yields, and late-stage functionalization of drugs and complex natural products. The visible-light-promoted dual catalytic method opens new avenues in direct C–H bond acylation and complements existing metal-catalyzed C–H bond functionalization methods (Scheme 3) [53].

**Scheme 4:**
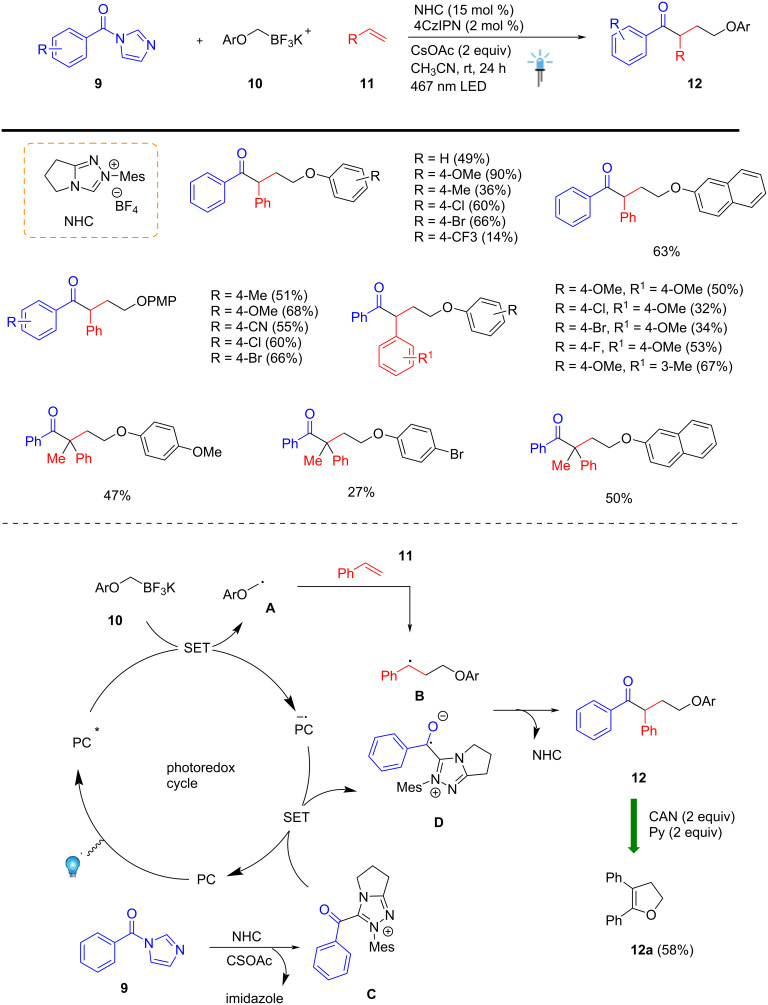
NHC/photoredox-catalyzed three-component coupling reaction for the preparation of γ-aryloxy ketones.

Recently, Scheidt et al. discovered an NHC/organic photoredox-catalyzed three-component coupling reaction for the efficient and novel preparation of γ-aryloxy ketone scaffolds **12**. This transformation builds on the emerging field of single-electron NHC catalysis by incorporating oxidatively generated aryloxymethyl radicals **A** as a key intermediate. A variety of γ-aryloxy ketones **12** were successfully prepared in the presence of NHC (15 mol %), photocatalyst (2 mol %), using 467 nm LED and a combination of alkene **11**, amide **9**, and aryloxymethyl potassium trifluoroborate salt **10** under mild conditions. The described catalytic system exhibited broad functional group tolerance and efficiently employed terminal alkenes **11** to generate quaternary centers adjacent to the carbonyl group. The key step in this organocatalytic process is the reduction of acylazolium **C** to generate a stable benzylic radical **D** is formed, and this extends the lifetime of the radical, allowing for radical–radical coupling reaction to afford the desired γ-aryloxy ketones **12** in good yields. The utility of this sustainable method was further demonstrated by the removal of the PMP protecting group using 2 equivalents of CAN and pyridine, affording 2,3-dihydrofurans **12a** in 58% (Scheme 4) [54].

**Scheme 5:**
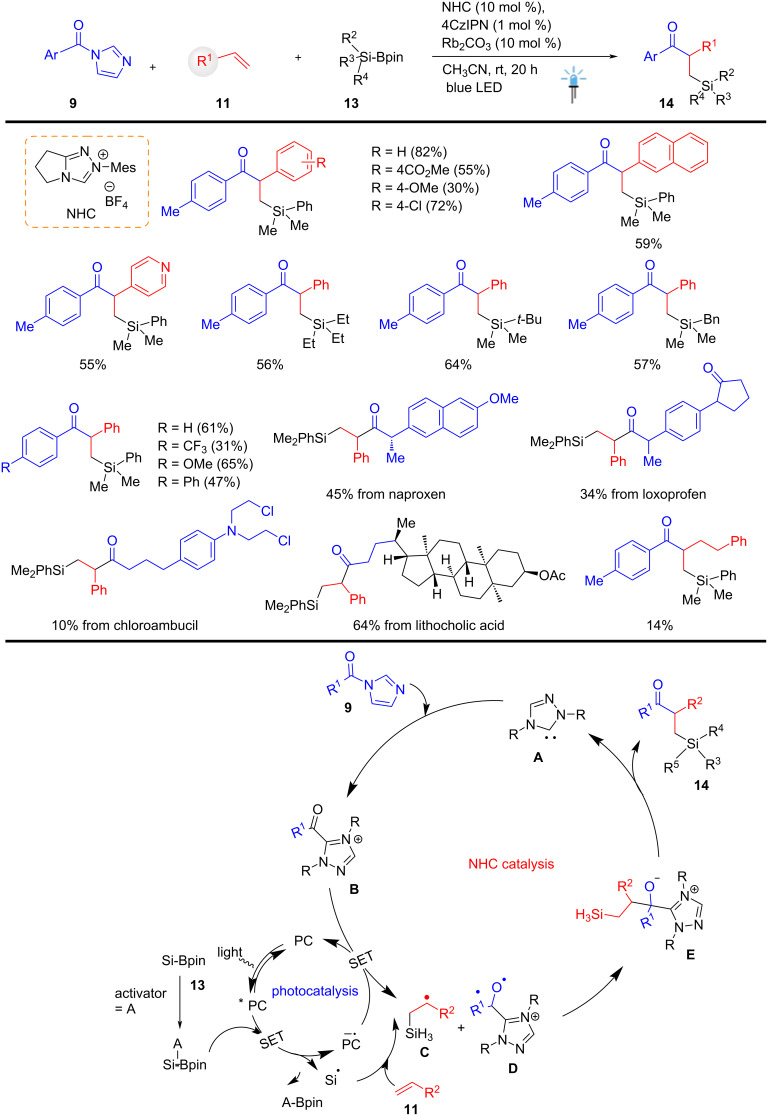
NHC-catalyzed silyl radical generation from silylboronate via dual catalysis.

In 2022, Sumida, Ohmiya, and co-workers developed a dual NHC/4CzIPN-organocatalyzed, versatile, light-driven silyl radical generation strategy from substituted silylboronates **13**. This photochemical method operates via catalytic activation of the silylboronate **13** in the presence of NHC (10 mol %) and 4C_Z_IPN (1 mol %) with Rb_2_CO_3_ (10 mol %) as a base in CH_3_CN at rt for 20 h. The approach affords highly functionalized β-silylated aryl ketones **14** in moderate to good yields, tolerates a variety of functional groups, and offers a wide substrate scope. Under the optimized conditions, Rb_2_CO_3_ provided better results than DMAP and Cs_2_CO_3_. The acyl azolium complex **B** was synthesized from acyl imidazole **9** using an NHC and Rb_2_CO_3_ as the base. Considering the redox potential of 4CzIPN and the acylazolium complex **B** (*E*_p_ *=* −0.81 V vs SCE), single-electron transfer (SET) reduction of **B** was thermodynamically feasible; however, the efficiency was found to be significantly low. The oxidation potential was measured to be around [*E*_p_ = +0.72 V] vs SCE, indicating that it is sufficiently positive to enable SET oxidation by photoexcited 4CzIPN*.* The authors described that the reduction potential of the photoredox catalyst (PC = 4CzIPN) was moderately low [*E*_red_(Ir^III*/^Ir^II^) = +0.66 V vs SCE in MeCN], and the silylboronate **13** permitted the formation of the silyl radical **C** under mild oxidation conditions. This visible-light-mediated dual catalytic method permits the efficient acylsilylation of substituted terminal alkenes **11** via NHC catalysis. Remarkably, this method afforded silyl radicals, which are typically difficult to prepare using conventional HAT-based catalytic methods. This method is used for late-stage modification of bioactive compounds, including natural products. These developments will find suitable applications in medicinal chemistry for introducible organosilyl groups (Scheme 5) [55].

A visible-light-induced Friedel–Crafts acylation of arenes and heteroarenes was reported by Studer et al., which belongs to the class of aryl and heteroaryl electrophilic substitutions and is a highly versatile synthetic transformation in traditional organic chemistry. A highly regioselective dual catalytic approach for the novel acylation of electron-rich benzo-fused arenes or heterocycles **15** was described. Aromatic C(sp^2^)–H bond acylation was achieved by dual catalysis through cooperative NHC and organophotoredox-catalyzed C–C cross-coupling of a benzo-fused aryl radical cation **C** with stable ketyl radical **B** as the key step. LED irradiation of photocatalyst leads to photoexcited PC*, which is reductively quenched by arene **16**, producing the corresponding aryl radical cation **C** and 3CzCIIPN^•–^ (*E*_1/2_ red = +1.56 V vs SCE). PC^•–^ then reduces the azolium ion **A** (*E*_1/2_ = −0.81 V vs SCE) to generate the ketyl radical **B**, thereby closing the photoredox catalysis cycle. 2-Methoxynaphthalene produced regioselective acylation products, but heteroarene substrates produced a mixture of products, for example, furan, thiophene, and benzothiophene.

**Scheme 6:**
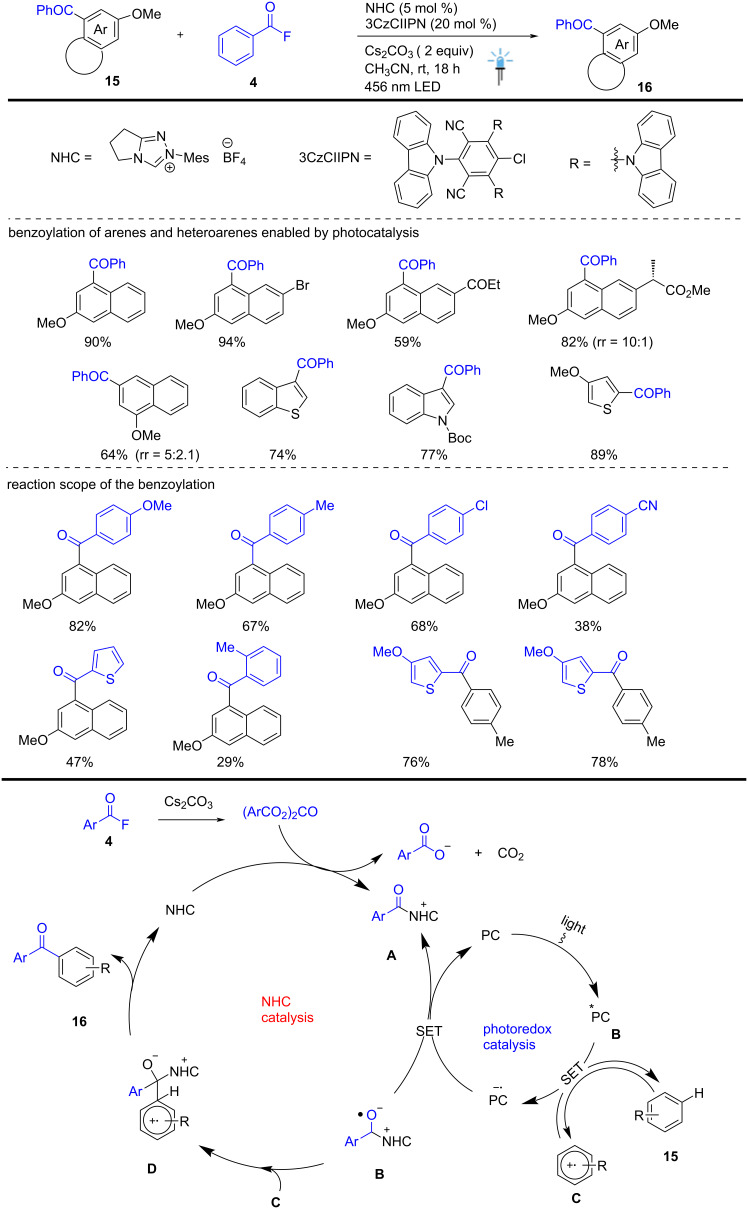
NHC-catalyzed C–H acylation of arenes and heteroarenes through photocatalysis.

Compared to the traditional LAs-mediated Friedel–Crafts acylation, the described radical method offers a regiodivergent result with good functional group tolerance. The Lewis acid-mediated traditional Friedel–Crafts acylation of 2-methoxynaphthalene typically proceeds at the *ortho* positions relative to the methoxy group due to the electron-rich nature of these sites, while the *meta* position remains deactivated under the ionic mechanism. Notably, the present work enables the highly regioselective formation of *meta*-acylated ketone products **16** through a radical pathway. The NHC-catalyzed novel acylation was achieved using acyl fluorides **4** and electron-rich arenes **15** in the presence of NHC (20 mol %), photocatalyst (5 mol %), and Cs_2_CO_3_ in CH_3_CN at room temperature for 18 h (Scheme 6) [56].

**Scheme 7:**
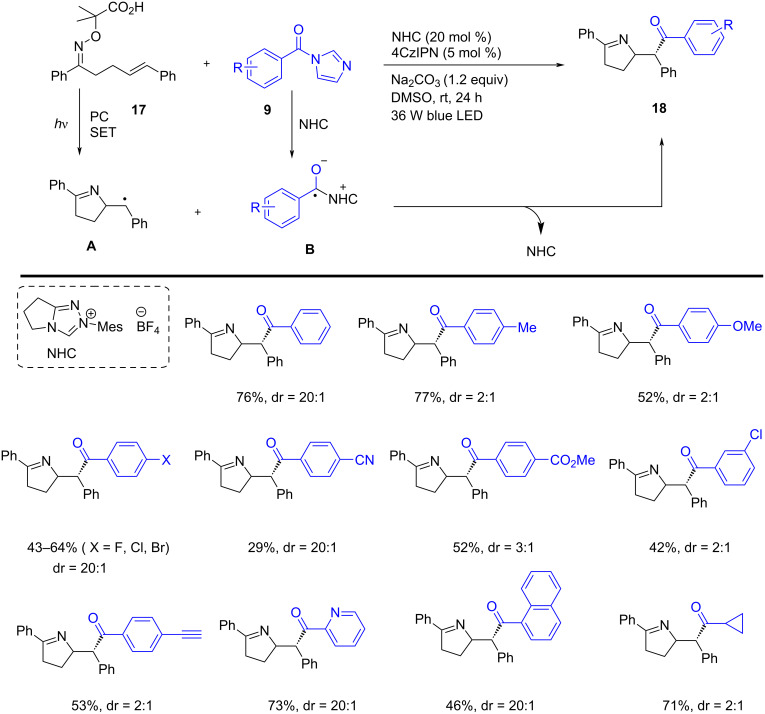
NHC-catalyzed iminoacylation of alkenes via photoredox dual organocatalysis.

Gao, Ye, and co-workers reported the iminoacylation of alkenes **17** via photoredox NHC dual catalysis in the presence of NHC (20 mol %), photocatalyst (5 mol %), with Na_2_CO_3_ (1.2 equiv) using a 36 W blue LED. In this method, the alkene-attached iminyl radicals **A** and the NHC-attached ketyl radical **B** are generated under photoredox NHC catalysis under visible-light irradiation. The 5-*exo*-trig radical cyclization of the alkene-tethered iminyl radicals **A** furnished a dihydropyrrole-derived radical coupled with the NHC group attached ketyl radical **B**. This approach features readily available starting materials, good functional group tolerance, and mild, transition-metal-free conditions. NHC-catalyzed radical approach furnishes access to substituted 3,4-dihydro-2*H*-pyrroles **18**, in good yields (Scheme 7) [57].

**Scheme 8:**
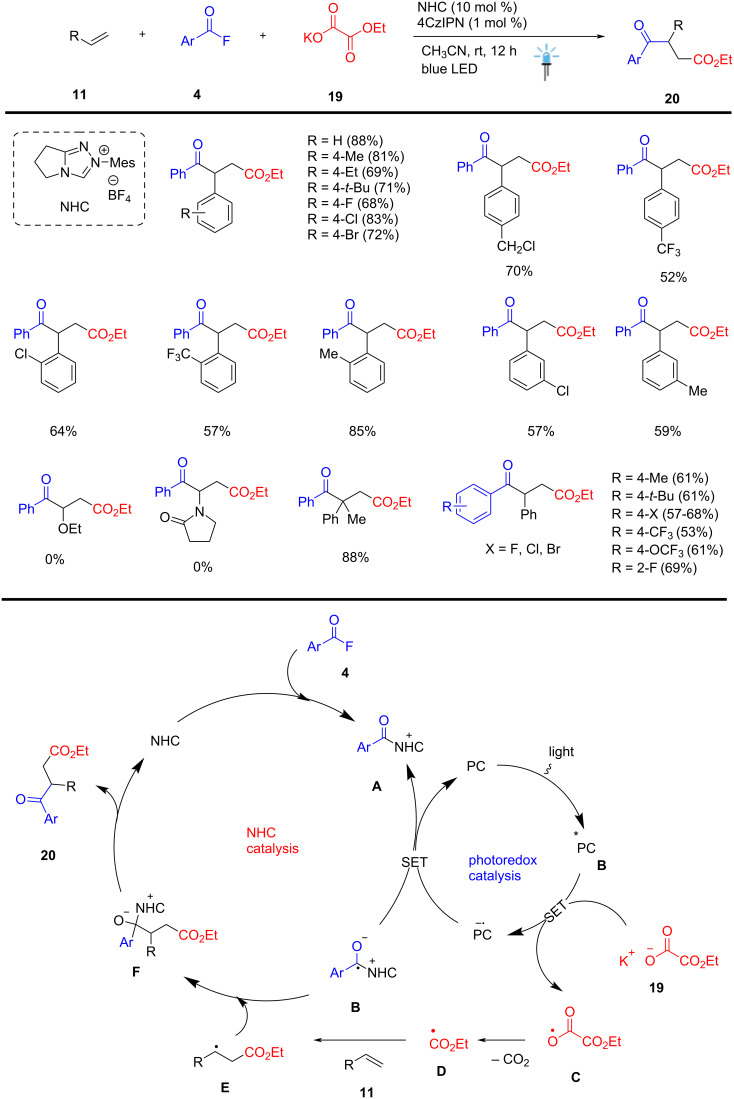
NHC/photoredox catalyzed direct synthesis of β-arylketoesters.

A novel strategy was discovered for 1,2-dicarbonylation of vinyl arenes **11,** acyl fluoride **4,** and keto-ester **19** via NHC/photoredox cooperative dual catalysis, which includes a radical cascade process reported by Zhu, Feng et al. The organic photoredox dual catalysis enables sequential C–C(O)OR and C–C(O)Ar bond formation, in the presence of catalytic amounts of NHC and organo-photocatalyst, leading to various β-aryl keto ester derivatives **20** in good to excellent yields. The key steps of the reactions undergo radical–radical cross-coupling with the alkyl ester radical **E** and benzyl radical **B** to afford the desired keto-esters **20** in up to 88% yield. This catalytic process displayed good functional group tolerance and broader substrate scope. The notable features of this dual catalysis approach are its mild reaction conditions, easy operation, and metal/base-free nature, which provide a valuable and facile method to rapidly access highly substituted β-aryl keto ester scaffolds **20** of interest in biological and materials sciences (Scheme 8) [58].

**Scheme 9:**
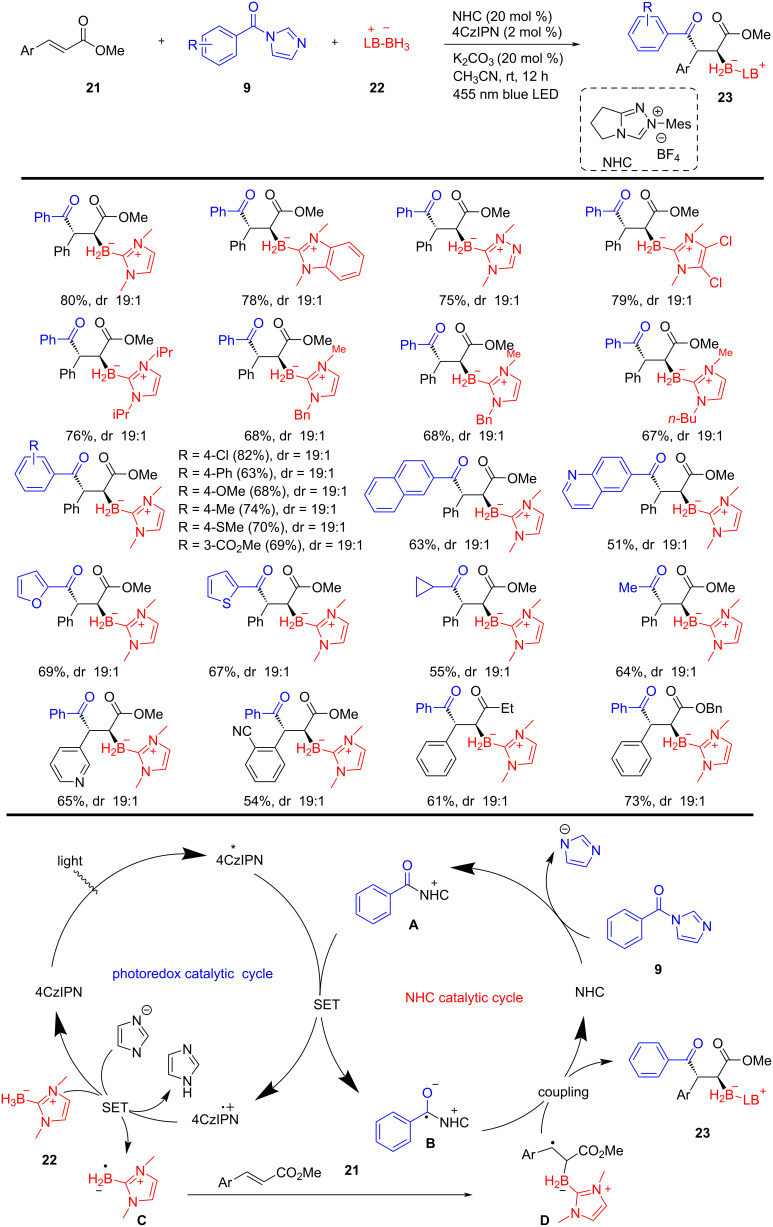
Visible-light-driven NHC/photoredox catalyzed borylacylation of alkenes.

In 2025, Xuan and colleagues, for the first time, developed a visible-light-driven dual catalytic method enabling the three-component borylacylation of substituted olefins **21**. This novel strategy allows the rapid and efficient preparation of boryl 1,4-dicarbonyl compounds **23**, valuable boron intermediates, from readily available starting materials and catalysts. The dual catalysis, merging NHC and photocatalysts, involves an radical species **B** and boryl radicals **D**, which undergo rapid radical–radical cross-coupling to yield the borylated 1,4-dicarbonyl compounds **23** in good yields. This system showed broad synthetic scope and excellent functional group tolerance of this multicomponent borylacylation, using NHC (20 mol %) and photocatalyst (2 mol %), in K_2_CO_3_ (20 mol %) under mild conditions, further highlighting the utility and versatility of this dual catalytic strategy (Scheme 9) [59].

### Organic dual catalysis enabled by visible-light-induced NHC and photoredoxcatalysts

**Scheme 10:**
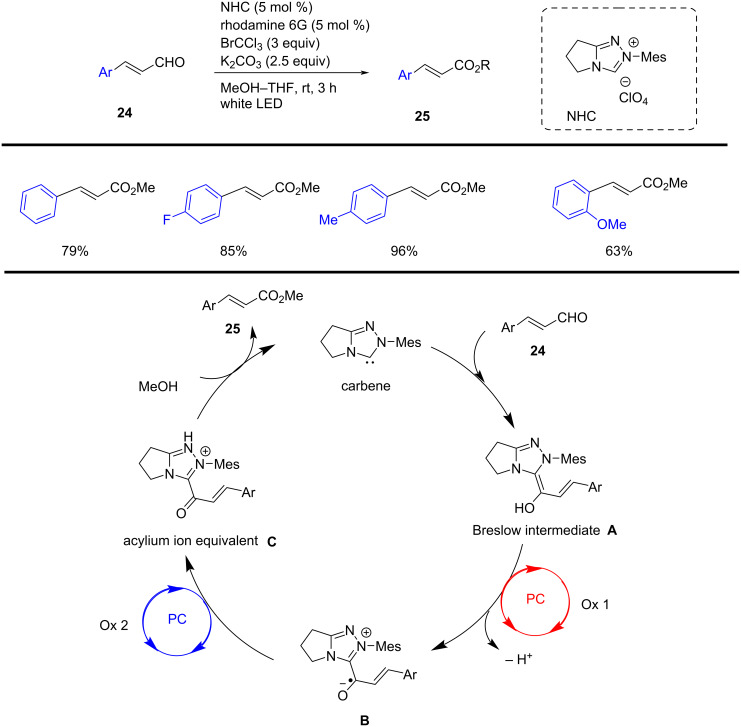
NHC-catalyzed oxidative functionalization of cinnamaldehyde.

In 2018, Miyabe et al. reported an innovative organocatalytic method for the oxidative esterification of functionalized cinnamaldehydes **24**. Dual photocatalysis, employing NHC (5 mol %) and rhodamine 6G (5 mol %), promoted the facile esterification of arylvinyl aldehydes **24** with BrCCl_3_ (3 equiv) and K_2_CO_3_ (2.5 equiv) in MeOH under visible-light irradiation. In contrast, oxidative esterification of the formyl group was also achieved via dual photocatalysis. Furthermore, various NHCs and photocatalysts were examined, and it was found that rhodamine 6G in combination with triazolium-based NHCs bearing mesityl substituents gave the best results compared to those bearing phenyl or pentafluoroaryl rings. Rhodamine 6G also offers the best outcomes compared to other photocatalysts such as fluorescein, alizarin red S, rhodamine B, and eosin Y. Since photoexcited organophotocatalyst rhodamine 6G (Rh6G*) possesses a sufficiently positive reduction potential in its singlet excited state S_1_ (*E*_red_* = +1.18 V vs SCE*). The reduced form of *Rh6G^•–^* subsequently reduces BrCCl_3_ to produce the CCl_3_ radical and Br^−^, due to its ground-state reduction potential (*E*_red_ = –1.14 V vs SCE in CH_3_CN), which is sufficiently negative for this conversion. In cycle 2, rhodamine 6G acts as a photoreductant, reducing BrCCl_3_ due to the adequate oxidation potential of its excited state (*E*_ox_* = –1.09 V vs SCE in CH_3_CN). The resulting oxidized *Rh6G^•+^*, with a ground-state oxidation potential of *E*_ox_ = +1.23 V vs SCE in CH_3_CN. This catalysis enables an efficient and straightforward photocatalytic preparation of functionalized aryl esters **25** through a radical pathway (Scheme 10) [60].

**Scheme 11:**
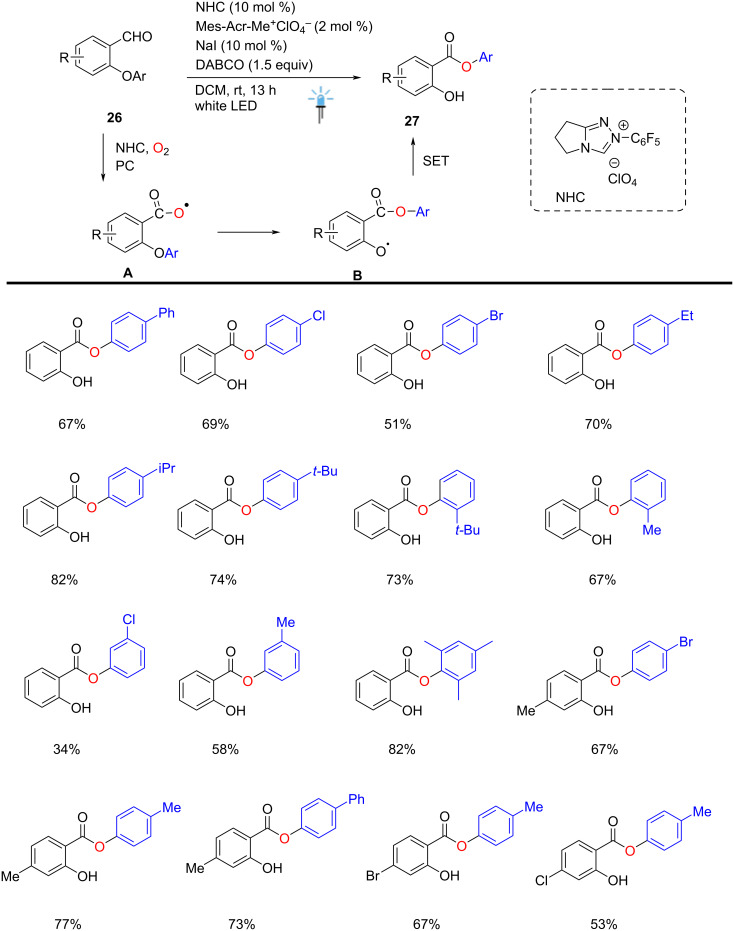
NHC/photocatalyzed oxidative Smiles rearrangement.

The NHC/organophotocatalyzed oxidative Smiles rearrangement of *O*-aryl aldehydes **26** has been developed using oxygen as the terminal oxidant under visible-light dual catalysis. This approach afforded highly functionalized 2-hydroxyarylbenzoates **27**, tolerating electron-deficient and electron-rich substituents. The C–O bond cleavage and the new C–O bond formation process were achieved using NHC (10 mol %), a photocatalyst (2 mol %), and DABCO (1.5 equiv), providing the corresponding aryl salicylates **27** in moderate to good yields. Mechanistic studies support the oxidation of the Breslow intermediate by oxygen in the presence of an acridinium photocatalyst and NaI (10 mol %) as a cocatalyst, affording an innovative method for oxidative carbene catalysis under mild conditions. Previous reports showed that 9-Mes-10-Me-acrydinium (*E*_red_^*^ in T1: +1.45 V vs SCE in CH_3_CN) exhibited more positive potentials, leading to an enhancement in chemical yields. One C–O bond was cleaved in this reaction while a new C–O bond was formed simultaneously using NHC and Mes-Acr-Me^+^ClO_4_^−^ in DCM at rt for 13 h, leading to the formation of aryl ester compounds **27** via radical-radical cross-coupling between intermediates **A** and **B** (Scheme 11) [61].

**Scheme 12:**
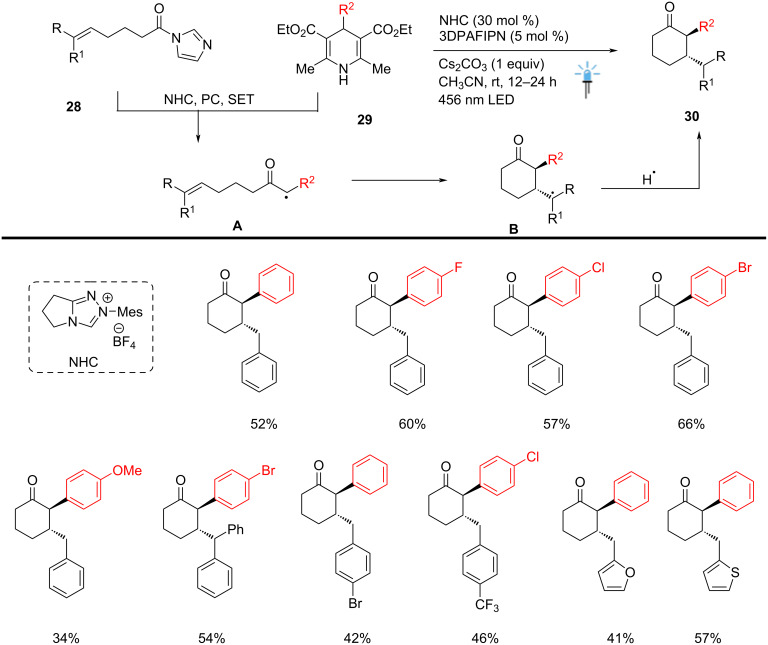
NHC-catalyzed synthesis of cyclohexanones through photocatalyzed annulation.

Scheidt and co-workers used a tandem annulation strategy, merging NHC and organic photoredox catalysis for the convergent novel synthesis of α,β-disubstituted cyclohexyl ketone scaffolds **30**. This cascade process rapidly forms two contiguous C–C bonds via a formal [5 + 1] cycloaddition. It represents a valuable approach for the α-functionalization of ketones using visible-light irradiation under mild reaction conditions. In a one-pot procedure, the reaction was carried out between alkenes **28** and coupling partner **29** in the presence of NHC (30 mol %) and a photocatalyst (5 mol %). Under these conditions organophotocatalyst (3DPAFIPN) was employed, significant amounts of cyclized product were observed and suggesting that the necessary redox potentials fall near its redox range (*E*_1/2_ PC*/PC^•−^ to *E*_1/2_ PC/PC^•−^ = +1.09 to −1.59 V vs SCE). This catalytic method provides an efficient route for synthesizing complex cycloalkanone scaffolds with potential applications in pharmaceutical and materials sciences (Scheme 12) [62].

**Scheme 13:**
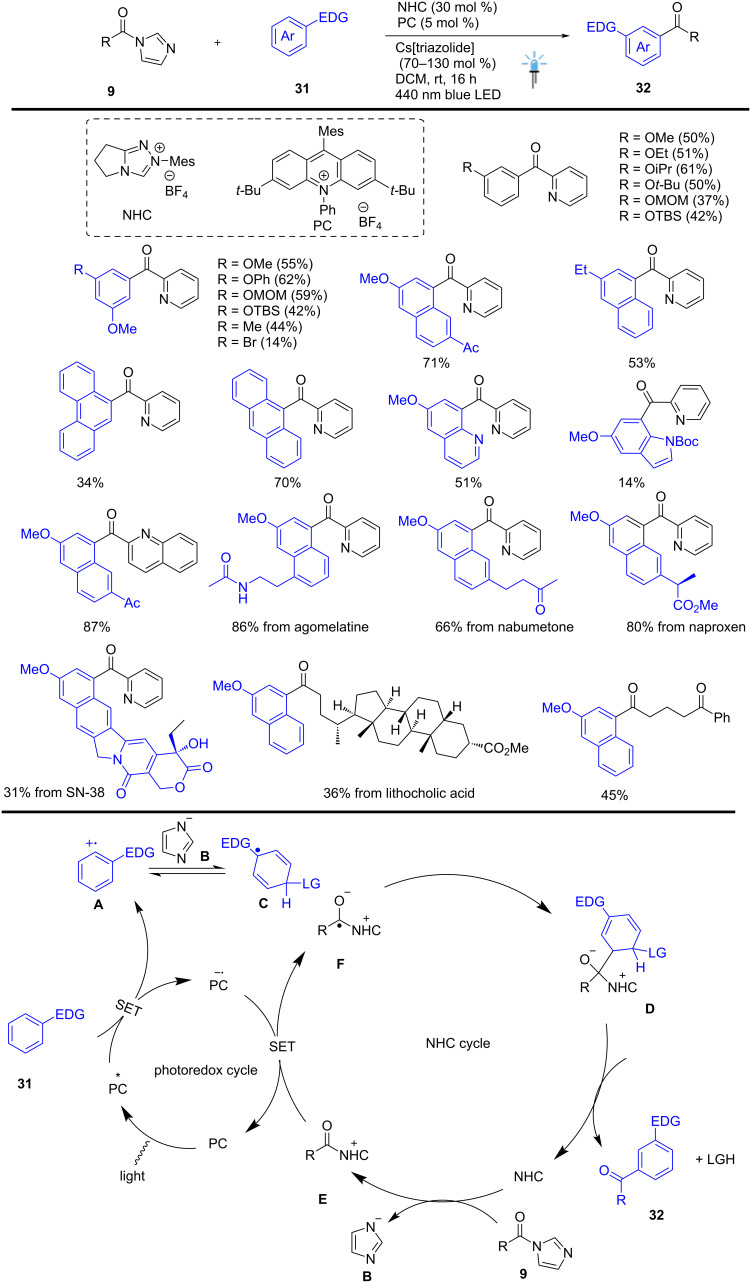
Dual organocatalyzed *meta*-selective acylation of electron-rich arenes and heteroarenes using blue LED.

Sumida, Ohmiya, and co-workers recently developed an NHC-and organic photoredox-catalyzed *meta*-selective Friedel–Crafts type acylation of electron-rich aromatic compounds **31**. The described catalytic system involves the efficient nucleophilic addition of an imidazolyl anion **B** to radical cation species **A**, generated via single-electron oxidation of electron-donating arenes **31**. The azolide anion **B** is released from acylimidazole **9** through an addition/elimination sequence in the presence of an NHC catalyst. The anticipated *meta*-acylation product **32** was achieved through highly selective C(sp^3^)–C(sp^3^)-radical–radical cross-coupling between the cyclohexadienyl radical **C** and the benzyl radical species **F**, formed via single-electron reduction. This photochemical method overturns the traditional *ortho* and *para*-selectivity observed in Friedel–Crafts acylation. The authors anticipate expanding this strategy to other reaction processes, allowing the efficient development of transformative synthetic methods, constructing new chemical spaces for drug discovery, and modifying bioactive molecules (Scheme 13) [63].

**Scheme 14:**
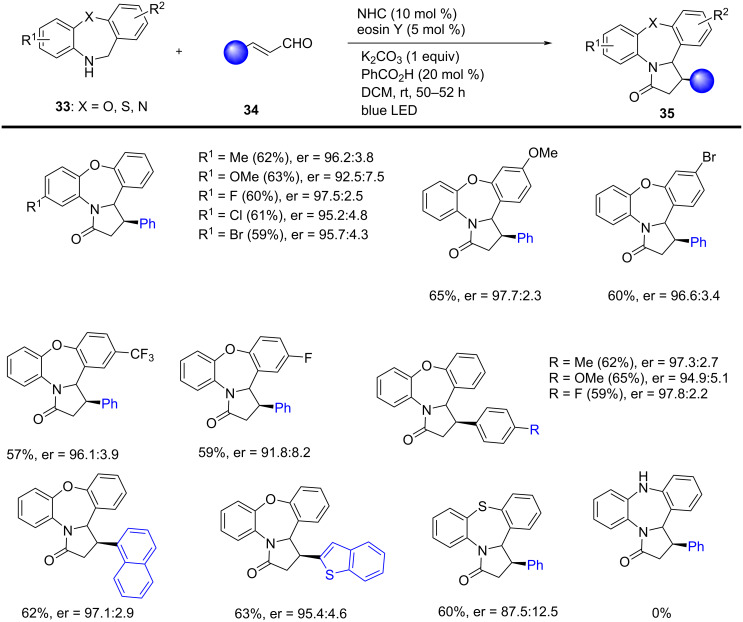
Asymmetric synthesis of fused pyrrolidinones via organophotoredox/*N*‑heterocyclic carbene dual catalysis.

Chauhan et al. developed a photocatalytic dual catalysis for an efficient stereoselective method that affords direct access to the pyrrolo[1,2-*d*][1,4]-oxazepin-3(2*H*)-ones **35** by merging organic eosin Y photoredox with carbene catalysis. Previous reports have shown eosin Y (*E*_red_^*^ in T_1_ [EY^−•^/EY^*^]: +0.83 V vs SCE in CH_3_CN), possessing less positive reduction potential, have shown the low catalytic activities of the reactions. This synergistic method permits the use of safer and non-toxic starting materials. In this relay catalytic strategy, a wide range of substituted enals **34** and dibenzoxazepines **33** worked well to achieve excellent enantioselectivities of the single diastereomers of the carbonyl products **35**. This reaction was carried out using NHC (10 mol %), eosin Y (5 mol %) under mild conditions. Various functionalized dibenzothioazepine substrates have also been employed to deliver the desired polycyclic compounds **35** in a moderate level of enantioselectivity (Scheme 14) [64].

## Conclusion

In conclusion, over the past two decades, organocatalyzed visible-light-promoted radical chemistry, particularly dual photocatalysis combining *N*-heterocyclic carbenes (NHCs) with organic photocatalysts, has emerged as a versatile and robust model for the sustainable synthesis of carbonyl groups and related valued organic compounds. This cooperative photochemical strategy offers broad functional group tolerance and operational simplicity. It aligns with green chemistry principles by operating under mild conditions, using sustainable methods with low-cost materials, and using non-toxic reagents. Its proven potential includes medicinal chemistry, pharmaceuticals, materials science, and the late-stage functionalization of complex bioactive molecules. Recent synthetic advances in methodology development, mechanistic understanding, and obtained results are expected to expand the applicability of NHC-photoredox dual or triple catalysis, enabling the efficient and eco-friendly synthesis of increasingly complex molecular architectures.

## Data Availability

Data sharing is not applicable as no new data was generated or analyzed in this study.
